# Using drones to transport suspected COVID-19 samples; experiences from the second largest testing centre in Ghana, West Africa

**DOI:** 10.1371/journal.pone.0277057

**Published:** 2022-11-01

**Authors:** Augustina Angelina Sylverken, Michael Owusu, Bernadette Agbavor, Alex Kwarteng, Nana Kwame Ayisi-Boateng, Patrick Ofori, Philip El-Duah, Richmond Yeboah, Sherihane Aryeetey, Jesse Addo Asamoah, Rita Ziem Ekekpi, Morrah Oppong, Richmond Gorman, Kofi Adjei Brempong, Emmanuella Nyarko-Afriyie, Felix Owusu Bonsu, Rita Larsen-Reindorf, Michael Rockson Adjei, Gifty Boateng, Franklin Asiedu-Bekoe, Badu Sarkodie, Dennis O. Laryea, Emmanuel Tinkorang, Patrick Kumah Aboagye, Anthony Nsiah Asare, Kwasi Obiri-Danso, Ellis Owusu-Dabo, Yaw Adu-Sarkodie, Richard Odame Phillips

**Affiliations:** 1 Department of Theoretical and Applied Biology, Kwame Nkrumah University of Science and Technology, Kumasi, Ghana; 2 Kumasi Centre for Collaborative Research in Tropical Medicine, Kwame Nkrumah University of Science and Technology, Kumasi, Ghana; 3 Department of Medical Laboratory Technology, Kwame Nkrumah University of Science and Technology, Kumasi, Ghana; 4 Department of Biochemistry and Biotechnology, Kwame Nkrumah University of Science and Technology, Kumasi, Ghana; 5 University Hospital, Kwame Nkrumah University of Science and Technology, Kumasi, Ghana; 6 Department of Medicine, Kwame Nkrumah University of Science and Technology, Kumasi, Ghana; 7 Institute of Virology, Charite, Universitätsmedizin Berlin, Berlin, Germany; 8 Zipline Mpanya Drone Site, Mampong, Ashanti Region, Ghana; 9 Ashanti Regional Health Directorate, Ghana Health Service, Kumasi, Ghana; 10 National Public Health Reference Laboratory, Ghana Health Service, Accra, Ghana; 11 Disease Surveillance Department, Ghana Health Service, Accra, Ghana; 12 Public Health Division, Ghana Health Service, Accra, Ghana; 13 Ghana Health Service Headquarters, Accra, Ghana; 14 Presidential Taskforce on COVID-19, Office of the President, Jubilee House, Accra, Ghana; 15 Department of Global and International Health, Kwame Nkrumah University of Science and Technology, Kumasi, Ghana; 16 Department of Clinical Microbiology, Kwame Nkrumah University of Science and Technology, Kumasi, Ghana; 17 Department of Medicine, Komfo Anokye Teaching Hospital, Kumasi, Ghana; Universitat Autònoma de Barcelona: Universitat Autonoma de Barcelona, SPAIN

## Abstract

**Background:**

The declaration of COVID-19 as a pandemic on March 11 2020, by the World Health Organisation prompted the need for a sustained and a rapid international response. In a swift response, the Government of Ghana, in partnership with Zipline company, launched the use of Unmanned Automated Vehicles (UAV) to transport suspected samples from selected districts to two foremost testing centres in the country. Here, we present the experiences of employing this technology and its impact on the transport time to the second largest testing centre, the Kumasi Centre for Collaborative Research in Tropical Medicine (KCCR) in Kumasi, Ghana.

**Methods:**

Swab samples collected from suspected COVID-19 patients were transported to the Zipline office by health workers. Information on the samples were sent to laboratory personnel located at KCCR through a WhatsApp platform to get them ready to receive the suspected COVID-19 samples while Zipline repackaged samples and transported them via drone. Time of take-off was reported as well as time of drop-off.

**Results:**

A total of 2537 COVID-19 suspected samples were received via drone transport from 10 districts between April 2020 to June 2021 in 440 deliveries. Ejura-Sekyedumase District Health Directorate delivered the highest number of samples (765; 30%). The farthest district to use the drone was Pru East, located 270 km away from KCCR in Kumasi and 173 km to the Zipline office in Mampong. Here, significantly, it took on the average 39 minutes for drones to deliver samples compared to 117 minutes spent in transporting samples by road (p<0.001).

**Conclusion:**

The use of drones for sample transport during the COVID-19 pandemic significantly reduced the travel time taken for samples to be transported by road to the testing site. This has enhanced innovative measures to fight the pandemic using technology.

## Introduction

In December 2019, the World Health Organization was informed about a cluster of patients who presented with pneumonia of unknown aetiology in the city of Wuhan (Hubei province) in China [1]. Shortly afterwards, a new type of coronavirus, now termed Severe Acute Respiratory Syndrome-Coronavirus-2 (SARS-CoV-2), was isolated and identified by Scientists from China [2]. The clinical spectrum of diseases caused by this virus, 2019 Coronavirus disease (COVID-19), has spread to all the World Health Organization (WHO) regions with a resultant enormous adverse global impact. Since then, it became a public health emergency of international concern and on March 11, 2020, the WHO declared it a global pandemic [3]. This action by WHO prompted an increased and sustained international action and response. As of March 4, 2022, over 440,000,000 cases were confirmed, with more than 6,000,000 deaths (https://www.worldometers.info/coronavirus/).

The African continent was not spared the scourge of the pandemic, with almost every country, including Ghana, reporting confirmed cases. The first confirmed case of COVID-19 in Africa was reported from Egypt on 14^th^ February 2020 [4] and in sub-Saharan Africa from Nigeria on 28^th^ February 2020 [5]. Ghana recorded its first confirmed case on 12^th^ March 2020 [6]. Following this, WHO recommended for all suspected cases of COVID-19 to be confirmed by reverse transcription-polymerase chain reaction (RT-PCR) [7]. By August 2020, South Africa and Ghana accounted for nearly half of the testing on the continent [8, 9]. In several African countries, the pandemic worsened the shortage of personal protective equipment and further revealed the vulnerability, ineffectiveness and weaknesses of many health care systems. One challenge during the pandemic was the shortage of COVID-19 RT-PCR test kits. While RT-PCR remains the gold standard, pre-analytical issues relating to transporting or shipping of samples from primary healthcare facilities to centralised testing laboratories was a challenge [10–12]. Ghana and other African countries had many other problems apart from the global logistical challenges [13–17]. For example, in Ghana, during the initial phase of the pandemic, there were only two medical/research laboratories with the capacity and expertise to analyse suspected COVID-19 samples by PCR. As such, samples from all rural and urban clinics and health centres which may be in far-flung areas with the two testing sites had their samples delivered to either one of these two sites. This meant samples had to be transported by road, which could take up to six days depending on how motorable the road was. Remote clinics that loathed to dispatch an ambulance for just one test would wait a few days to collect enough samples to make the trip worthwhile and cost- effective. Such a decision ended up prolonging patients’ anxiety and delaying the contact tracing protocols necessary to mitigate the spread of the virus. As expertly stated by the Director of the Africa Centres for Disease Control, Dr. John Nkengasong, “If you don’t test, you don’t find”, this means that testing is the most important thing, and whatever it takes to make it faster, makes it better [18].

On April 1, 2020, after confirming 834 COVID-19 cases, Ghana adopted Unmanned Automated Vehicles (AUV), popularly known as Drones, to make regular long-range deliveries into densely populated urban areas in the fight against COVID-19 [19]. There was an agreement with Zipline [20], the operators, to transport suspected COVID-19 samples from two of their main drone zones. Located in Mampong in the Ashanti region and the Omenako site in the Eastern region of Ghana, the drones transported samples to the two main testing centers; Kumasi Centre for Collaborative Research in Tropical Medicine (KCCR) in Kumasi located in the middle belt of Ghana and Noguchi Memorial Institute for Medical Research (NMIMR) in Accra, which is in southern Ghana covering distances of over 100 km in the shortest time. In this paper, we present experiences and the impact of the drone technology on the time taken for sample delivery in response to Ghana’s COVID-19 laboratory diagnosis in the second-largest testing site, the KCCR, in Ghana, a West African country.

## Materials and methods

### Study sites

Data were collected from April 2020 to June 2021 at KCCR. During the early phase of the pandemic, the laboratory technicians at KCCR received samples from 12 out of the 16 regions in Ghana and tested approximately 1,200 samples daily. Samples were transported to the Centre by road using dedicated cars, ambulances, motorcycles, and courier services. The use of drones was introduced for sample delivery at KCCR on April 16, 2020.

Zipline has two main sites in Ghana for transporting COVID-19 suspected samples; the Mpanya and Omenako drone sites (www.flyzipline.com). The Mpanya site is located at Mampong in the Asante Mampong Municipal Assembly in the Ashanti region. The site is bounded to the south by Sekyere South district, to the east by Sekyere Central and to the North by Ejura-Sekyedumase districts. Closer to these districts are the Sekyere Kumawu and Sekyere Afram Plains and to the North, Ejura-Sekyedumase is bounded by the Atebubu Amantin District in the Bono East Region. Other districts that access the Zipline drone service include the Techiman Municipal health directorate, Pru East District, and the Nkoranza South district health directorates, all in the Bono East Region. Samples were sent from all these districts to KCCR.

The Mpanya Zipline drone site boasts of multiple drones for transportation of suspected COVID-19 samples. Before the pandemic, The Ghana Drone Delivery Service was launched on 24 April 2019 [21] with aims to deliver medical supplies such as blood, plasma, protective equipment, medicines and vaccines [21, 22] within designated areas.

**Fig 1:**
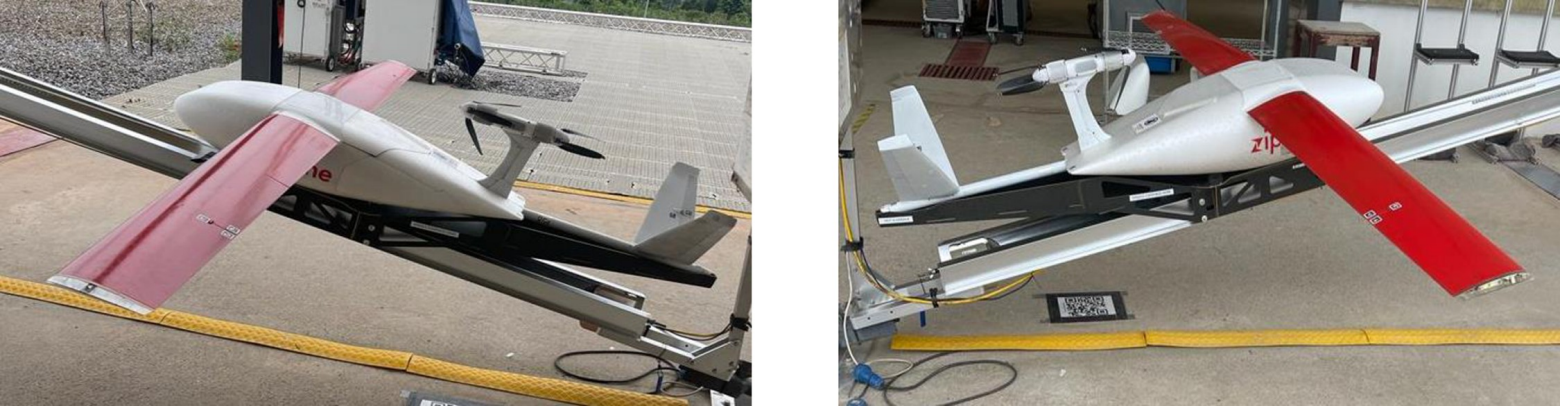
Pictures of some of the drones used by Zipline (pictures taken by Bonsu F.O).

The Zipline drones are fixed-winged with dual propellers with a speed of 100km/hr. Made of three parts (body or belly, wings and rechargeable batteries), the facility boasts of several of these parts which are assembled depending on the demand for use. This means that at a point in time several drones can be assembled for use. Again, depending on the quantum of samples to be transported, an appropriate belly or body which can accommodate the package size is used for transportation (Fig 1).

### Sample collection and transportation

Nasopharyngeal and or oropharyngeal swabs were collected from suspected individuals or their contacts from hospitals and health centres located in communities close to the Mampong Zipline Mpanya drone site. Sample collection followed WHO protocols [23]. Samples were transported to the Zipline office following the principles of packaging techniques and transportation protocols for infectious biological samples [24] which have been adopted by the Ghana Health Service. Before samples were transported, a request was made by the hospital to the Zipline office by the Disease Control Officers in the respective districts.

At the Zipline office, trained personnel upon receipt of the samples, repackaged them into bio-safe containers and placed them into the drone bellies. Included in the package was an invoice that contained the name of the sending district or hospital, number of samples and sample codes. Sample codes were generated by Zipline. The package was accompanied by a barcoded case investigation form designed by the Ghana Health Service for collecting data on COVID-19 suspected cases.

### WhatsApp platform

A social media group platform ‘[Zipline] KCCR’ was created on WhatsApp (a social media application) on 16^th^ April 2020. There are two (2) representatives from each of the districts from where samples were to be sent, four (4) representatives from the Regional Health Directorate, Ashanti Region and Bono East and two representatives from Zipline. At KCCR, two COVID-19 testing team leads and six (6) laboratory technicians are also members of the WhatsApp group.

**Fig 2:**
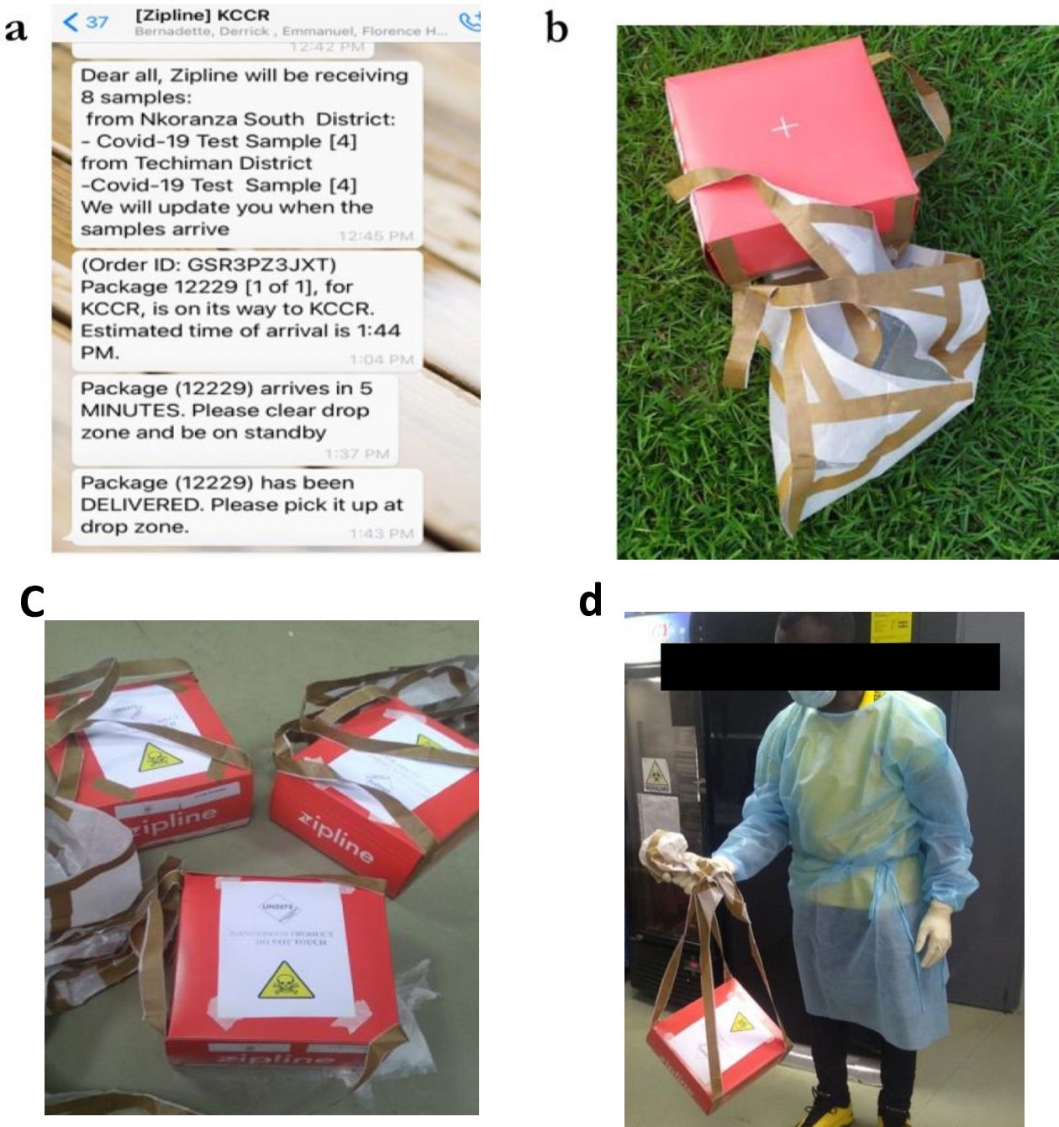
**a**: WhatsApp chat message with information on samples being sent to the testing Centre; **b**: Package with sample at the drop zone yet to be picked up by testing team **c**: Sample packages transported to the laboratory for analysis **d**: Sample receiving team member transporting sample to the laboratory.

The WhatsApp group was used to inform the KCCR team when samples were sent. Only messages related to suspected COVID-19 samples sent were posted on the WhatsApp page. Each message sent contained the number of samples and the name of the sending district. Notification on the order identification (ID), and the estimated time of arrival were provided. An additional message was sent when the package was 5 minutes away from delivery. The final message was sent when the package dropped in the drop zone. The group was administered by Zipline representatives (**Fig 2A**).

The drones dropped the samples at a designated drop zone at KCCR. Delivery was effected from a high elevation, but all bio-containers landed safely because of their specially designed attached parachutes. Members of the testing team in charge of receiving samples and documentation picked the package up for processing in the designated laboratories (**Fig 2B–2D)**.

Upon receipt, sample numbers and their means of identification were cross-checked with the list provided and the information logged into the sample reception system at KCCR. All districts whose samples were tested by the laboratory were assigned codes for easy identification, as shown in **Table 1**. Information from the log was entered into Microsoft Excel (Microsoft Corporation, Redmond, WA) and analysed. Proportions were generated using Excel. Comparison of the distances covered, and time taken for sample delivery using roads and drones was done using the student T-test on GraphPad Prism version 5.0 (GraphPad Software, Inc., La Jolla, CA). Alpha values < 0.05 were considered statistically significant.

**Table 1:** District names and codes for samples sent via Zipline.

District/Municipal Name	Assigned Code
Asante Mampong	ASM
Ejura Sekyere Odumase	EJS
Sekyere South	SES
Sekyere Central	SEC
Sekyere Afram Plains	SAP
Sekyere Kumawu	SEK
Atebubu Amantin	ATA
Techiman	TEC
Nkoranza South	NKS
Pru East	PRE

### Ethical approval

Ethical approval for this study was obtained from the Committee on Human Research, Publication and Ethics (CHRPE) of the School of Medicine and Dentistry, Kwame Nkrumah University of Science and Technology (KNUST) (CHPRE/AP/566/21) and the Ethical Review Committee of the Ghana Health Service (GHS-ERC087/03/20).

## Results

### Number of samples received via drone

From April 2020 to June 2021, a total of 2537 suspected COVID-19 samples were received via Zipline drones (S1 Table). These samples were sent from ten (10) districts in the Ashanti and Bono East regions through a total of 440 deliveries from the Zipline Mpanya drone site at Mampong. Ejura Sekyere Odumase Municipal health directorate transported the highest number of samples (765) making up 30% of the total number of samples received within the reporting period. The least number of samples received, fifteen (15), was from the Pru East District in the Bono East region in 4 deliveries.

**Fig 3:**
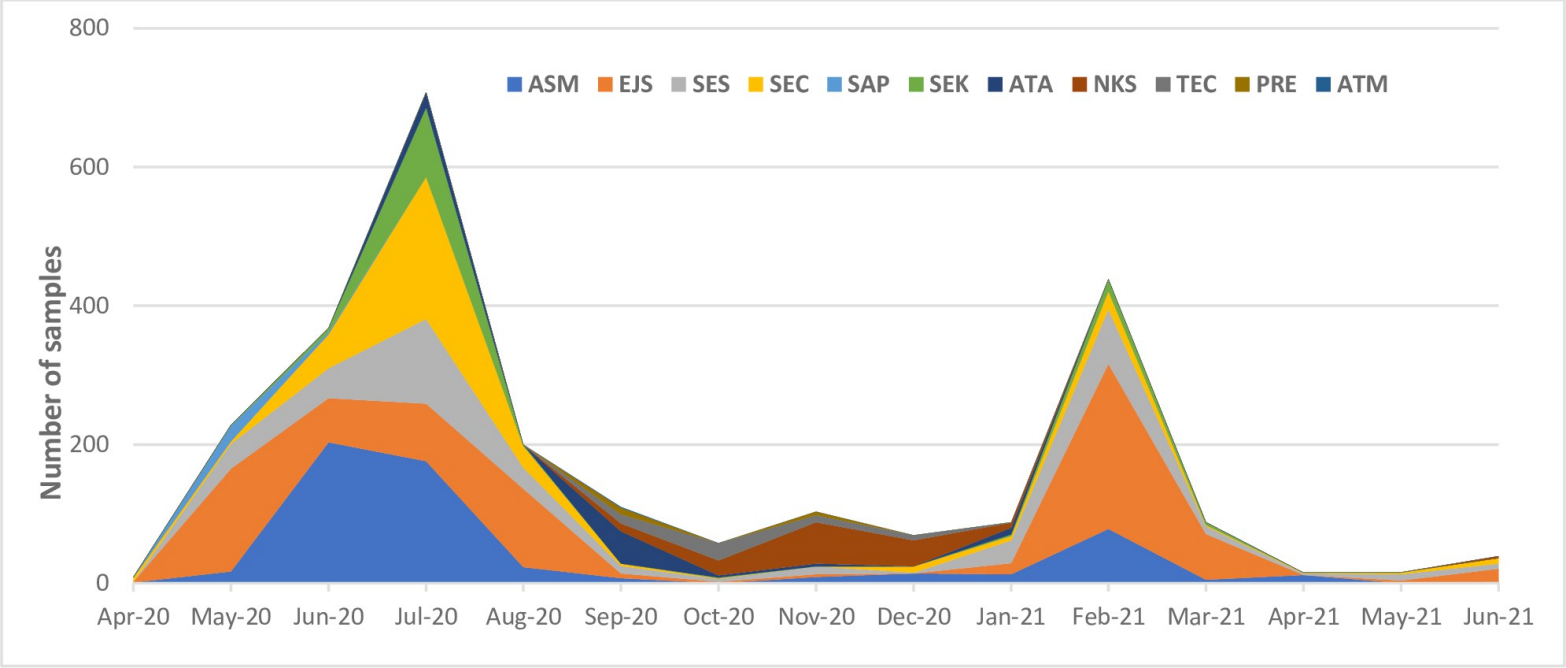
Distribution of samples received via zipline per district (ASM-Asante Mampong; EJS-Ejura Sekyere Odumase; SES-Sekyere South; SEC-Sekyere Central; SAP- Sekyere Afram Plains; SEK-Sekyere Kumawu; ATA-Atebubu Amantin; TEC-Techiman; NKS-Nkoranza South; PRE- Pru East).

Overall, the number of samples received corresponded with the rising number of cases during the two main waves that hit the country. Samples peaked in July 2020 (708) and February 2021(438) in 91 and 50 deliveries, respectively (**Fig 3**).

### Positivity

Of the 2537 samples received from the 10 districts within the study period, a total 560 (22%) tested positive for SARS-CoV-2 (Table 2). Of the total positive samples, the highest was received from Sekyere South (161; 40.25%).

**Table 2:** Positivity rate of samples received.

District	Number of samples sent	Number of positives	% Positivity
ASM	560	144	25.71
ATA	87	11	12.64
EJS	765	133	17.40
SAP	27	1	3.70
SEC	352	78	22.20
SEK	134	30	22.40
SES	400	161	40.25
NKS	141	0	0.00
PRE	15	0	0.00
TEC	56	2	3.60
**ALL**	**2537**	**560**	**22.10**

### Distance/time relations

During the period, the farthest district to transport samples to KCCR via Zipline drones was Pru East. The distance from the Pru East district to KCCR in Kumasi was 270 Km and to the Zipline office at Mpanya was 173 km. **Table 3** shows a comparison between the distances from the districts to the laboratory and the drone zone. There was no statistically significant difference between the distances covered by the districts to KCCR and to the Zipline office (p = 0.1085). On the contrary, there was a significant difference between the estimated times of arrival (ETA) to KCCR and to the Zipline Office when the ETA to KCCR was compared to the ETA to Zipline (p = 0.0237). Averagely, it takes 96 minutes to travel from the Zipline office to the KCCR by road covering a distance of 63 km.

**Table 3:** Distances covered by districts to the Zipline Mpanya office and to KCCR.

District Codes	Distance to KCCR (kilometre)	ETA to KCCR (Minutes)	Distance to Zipline (kilometre)	ETA to Zipline (minutes)
ASM	67.4	101	15	7
ATA	161	205	100	88
EJS	106	136	47	40
SAP	54	90	32	77
SEC	59	98	11	13
SEK	50	84	36	52
PRE	270	316	173	188
NKS	126	187	92	120
TEC	154	215	99	132
SES	49	76	22	30

ETA: Estimated Time of Arrival; KCCR: Kumasi Centre for Collaborative Research in Tropical Medicine

Table 4 provides a comparison of time taken for delivery of sample by road and by drone. On average each of the 10 districts travelled 110 km to transport samples by road to the KCCR laboratories while it took an average of 62.7 km (11–173 km) to the Zipline office at Mampong. Comparatively, it took significantly shorter time average 39 minutes (36.50–43.30 minutes) to transport samples by drones to KCCR from the Zipline office in contrast to an average of 117 minutes (76–316 minutes) from the various districts to KCCR by road (p< 0.0001 Student T test).

**Table 4:** Comparison of time taken for sample delivery by road and by drone.

		Time taken for sample delivery				
District	Distance to KCCR (km)	Mean time by drone (Min)	Mean time by road (Min)*	Mean Difference ±SEM	95% CI	p-value
ASM	67	39.70	101	61.31 ± 0.73	59.87–62.76	<0.0001
ATA	161	36.50	205	168.5 ± 0.81	166.8–170.2	<0.0001
EJS	106	39.00	136	97.04 ± 0.56	95.93–98.15	<0.0001
SAP	54	40.80	90	49.18 ± 0.99	47.10–51.26	<0.0001
SEC	59	39.00	98	59.00 ± 0.71	57.60–60.40	<0.0001
SEK	50	38.70	84	45.32 ± 0.89	43.53–47.11	<0.0001
SES	270	38.80	76	37.50 ± 0.88	35.77–39.24	<0.0001
NKS	126	37.60	187	149.4 ± 1.15	147.1–151.7	<0.0001
PRE	154	43.30	316	272.8 ± 1.97	267.9–277.6	<0.0001
TEC	49	38.20	215	176.8 ± 1.22	174.3–179.3	<0.0001
ALL (Averages)	110^a^	39.00^b^	117^c^	77.83 ± 2.07	73.76–81.90	<0.0001

a-Average distance covered from the districts to KCCR, b- Average time taken by drone from Zipline office to KCCR, c- Average ETA to KCCR by road,

*-ETA was used in the computation.

## Discussion

The hallmark of effective disease control is early diagnosis, and the outcome of Africa’s recent outbreaks before COVID-19, especially Ebola virus and Lassa fever, largely depended on this. In the case of COVID-19, early diagnosis is even more important, considering the dire transmission risks potentially posed by asymptomatic individuals [25]. Therefore, it was important to deploy a strategy for the rapid transportation of suspected samples to testing sites for prompt testing. Such a prompt action is needed to implement contact tracing and, importantly initiate isolation procedures for positive cases.

In a swift response to the pandemic containment and the call for more testing, Ghana became the first country to introduce the drone technology in sample transport to some testing sites [26, 27]. On the 18^th^ of April, 2020, KCCR received its first test sample from the Sekyere Central District, and subsequently, other districts followed. Ghana, like most African countries, is bedevilled with poor road network [28]. Considering that depending on the nature of the road, the transport of samples that would have taken about 5 hours covering distances over 200 kilometers had been reduced to about 40 minutes was a great deal for the health workers. Not only did it save time, it also helped these smaller districts conserve resources, both human and material, which would be used to manage other disease conditions and to support other aspects of the health system in the surrounding communities.

Proximity to the Zipline office may have also influenced the number of samples received from a district as is reflective of the Asante Mampong Municipal Assembly, where the Zipline office is located, transporting a total of 560 samples (22% of total samples received). Other districts including Sekyere Central and Sekyere South which are located less than 30km from the Zipline office also transported 352 (14%) and 400 (16%) samples, respectively. On the contrary, the farthest districts, Pru East and Atebubu Amantin, transported less than 100 samples forming less than 2% of total samples received via drone transport. The distance to the drone office may still be a reason for some districts to accumulate samples to transport in order to avoid several trips times to the drone office. A possible suggestion will be that there is the need for more drone offices to be situated in the districts particularly those that are at the periphery and far off from the major cities where the drone sites and offices are located.

The high numbers received from some districts may also reflect the infection rate and the high positivity rates in those districts as compared to other districts. The use of drones to transport the samples has saved a lot of resources and time that potentially could be used for other activities such as contact tracing and for effective and rapid response to management protocols. The use of the drones during the pandemic has also increased the visibility and the ability of Ghana to employ among other technologies [19, 26, 29], this new technology in emergency response. This will enhance the ability of Ghana to meet the general goal of universal health coverage for all which is enshrined in the United Nations Sustainable Development Goals (SDGs).

Globally, the role of drones during this pandemic has been limited to the transport and delivery of essential supplies including medical supplies, mostly personal protective equipment as reported in China and India as well as the USA [30, 31], dissemination of information [18], food supplies, aerial and mass disinfection of public places and crowd surveillance [18, 19]. Critically, drones have also been employed in mass screening with infrared cameras used to measure the temperature of the masses [18]. In Africa, the onset of the pandemic revolutionalised health systems and posed a transformative shift in drone services in healthcare [32]. Before the pandemic, some African countries had already incorporated drones in military, agriculture and partially in healthcare systems. In Rwanda, drones were used to promote awareness, transport blood samples and other essential medications to remote clinics [33, 34]. Outside sub-Saharan Africa, drones were highly utilized in collecting and analysing COVID-19 data [35]. Drones have also been used in disinfecting contaminated objects and surfaces, broadcasting, surveillance and delivering essential products [36, 37]. They have also been used in monitoring social distancing. and delivery of goods [38] medical supplies [39, 40] and sometimes samples [41]. Very few reports were available in favour of its use to transport suspected COVID-19 samples for testing especially in West Africa. Ghana, therefore, stood tall when this initiative was taken and executed. Even though this technology was introduced in the country in April 2019, it was restricted to the transport of medical supplies and medicines particularly for emergencies including blood and blood products. During the pandemic, it came in handy since the service providers were already well-established and only had to add the transportation of suspected COVID-19 samples to its line of activities. Indeed, the gains obtained from the use of this technology outweigh the losses.

There were however, a few challenges that were identified, most importantly with the weather. Even though there are drones designed specifically for weather and other climate studies [42, 43], the ones used for sample transport were not equipped with such technology. It is known that such Unmanned Automated Vehicles (UAVs) have limited access to weather information especially that related to visibility, winds and even rains which can lead to the destruction of the electrical systems, drones being blown off- course or even crashed.

At the peak of the pandemic in the country was the rainy season and its associated weather conditions which affected the operations of the drones a couple of times. Results of these unfavorable weather conditions impacted the activities of the Zipline company by either delaying the sample delivery by a day or a few hours, otherwise, samples were hand-delivered via land transport. In such a situation, notification was made on the WhatsApp platform. In spite of these challenges, the use of drones for the delivery of samples was very effective and considered a great technological breakthrough in the pandemic.

The importance of this technological innovation is reflected in the travel time it saves health workers in transporting samples and resources to be used. To this end, some health workers have expressed satisfaction in the drone services and are relieved to know that sample transport, which would have taken about one-third of their time, had been reduced [44]. It also increased the number of samples to be tested in a shorter time. One important benefit was the reduction in the human contact among health workers transporting samples and laboratory staff receiving samples, thus increasing social distancing, which is one of the generally accepted preventive guidelines for COVID-19 [45, 46]. Overall, there was a significant reduction in the transportation time for districts located close to the drone office. Since this may offer less benefits for districts far away from the drone office, it will be important to have more drone sites situated closer to these districts in order to benefit from reduced transportation times. Beyond siting the drone offices and services closer to these districts, it is the hope that the drone services will be extended beyond the pandemic to cover other diseases that need to be tested elsewhere [47].

## Conclusion

The SARS-CoV-2 pandemic has reiterated the need for technological advancements to combat it and many individuals, corporate organisations and governments responded appropriately including the use of drones. Instead of waiting several days for a batch of samples to be transported, coupled with numerous challenges such as bad roads, a single test from a rural area can be delivered for analysis in less than an hour. Especially for districts close to the drone centre, using contactless drone delivery to transport COVID-19 test samples made it possible for tests to be run in time, allowing the government to respond to the pandemic and enhanced potential to save lives.

## Supporting information

S1 Table Names of health facilities within the districts. A list of all the health facilities under each district that transported samples via drone.
(DOCX)
