# Supplementary material for: Using drones to transport suspected COVID-19 samples; experiences from the second largest testing centre in Ghana, West Africa
Source: PLoS One. 2022 Nov 1;17(11):e0277057. doi: 10.1371/journal.pone.0277057 (PMC9624400; doi:10.1371/journal.pone.0277057)
Supplement: S1 Table — A list of all the health facilities under each district that transported samples via drone. (DOCX) [file pone.0277057.s001.docx]

S1 Table: Names of health facilities within the districts

A list of all the health facilities under each district that transported samples via drone

| **District (Code)** | **Health facilities** | **No of samples submitted** |
| --- | --- | --- |
| Asante Mampong (ASM) | Mampong Government Hospital  Seventh-Day Adventist Clinic  Asante Mampong District Health Hospital  University clinic (UEW)  Kofiase Health Centre  Calvary Health Centre  Nkwanta CHPS  Quality Health Care  Asaam Health Centre | 451  82  15  5  2  2  1  1  1 |
|  |  | **560** |
| Ejura Sekyere Odumase (EJS) | St. Luke Hospital, Kaase  Aframso Health Centre  Ejura Government Hospital  Ejura District Health Hospital  Sekyeredumase Polyclinic  Homaka Community Health and Planning Services (CHPs)  St. Anthony Ann Hospital, Donyina  Ejura Community Hospital  Ejura District Health Directorate  Ejura Market Centre | 171  3  467  3  21  4  6  1  87  2 |
|  |  | **765** |
| Sekyere South (SES) | Salvation Army Hospital, Wiamoase  Wiamoase SDA Hospital  Agona Government Hospital  SDA Hospital, Asamang  Sekyere South District Health Directorate  Pope John Paul II Medical Centre  Jamasi Health Centre  Kona Health Centre  Boanim Health Centjre  Sacred Heart Health Centre  Divine favour hospital  Ebeyie CHPS  Domeabra | 54  46  157  28  17  49  30  3  1  4  5  5  1 |
|  |  | **400** |
| Sekyere Central (SEC) | Sekyere Central District Health Directorate  Kwamang Health Centre  Beposo Health Centre  Nsuta Health Centre  Jeduako Health Centre  Abordease Health Centre  St Vincent Clinic | 207  100  31  9  1  2  2 |
|  |  | 3**52** |
| Sekyere Afram Plains (SAP) | Auntie Riek Clinic  St. Vincent De Paul Clinic | 24  3  **27** |
| Sekyere Kumawu (SEK) | Sekyere Kumawu District Health Directorate  Kumawu Polyclinic  West Phalian Medical Centre  Bodomase Health Centre  Woraso Health Centre | 58  69  4  1  2 |
|  |  | **134** |
| Atebubu Amantin (ATA) | Amanten Health Centre | **87** |
| Techiman (TEC) | Holy Family Hospital | **56** |
| Nkoranza South (NKS) | Unknown  Nkoranza Health Centre  Nkoranza South Municipal Health Directorate | 68  62  11 |
|  |  | **141** |
| Pru East (PRE) | Pru East District Health Directorate | 15 |
|  |  | **2537** |
